# Supplementary material for: Advances in macrophage and T cell metabolic reprogramming and immunotherapy in the tumor microenvironment
Source: PeerJ. 2024 Jan 15;12:e16825. doi: 10.7717/peerj.16825 (PMC10795528; doi:10.7717/peerj.16825)
Supplement: Supplemental Information 1 [file peerj-12-16825-s001.docx]

Table S1. Metabolism and function of major immune cells in TME

| Granulocyte | Neutrophil | Neutrophils are the most abundant myeloid cells in human blood and have both pro- and anti-cancer effects. Among them, pro-carcinogenic effects are related to their release of ROS leading to oxidative DNA damage in the lungs, and also support tumor cell proliferation through various paracrine signaling pathways; anticancer effects are related to induction of tumor cell detachment, release of nitric oxide, and uptake of plasma membrane mediated by Fc. |
| --- | --- | --- |
|  | Eosinophil | Eosinophils produce damaging proteins that play an important role in allergic reactions. When eosinophils are exposed to IFN-γ and TNF-α in the TME, they release large quantities of chemokines that recruit anticancer T cells, exerting antitumor effects, and may also contribute to tumor formation, including by promoting the progression of inflammation. |
|  | Mast cell | Mast cells can promote inflammation, inhibit tumor cell growth, and induce tumor cell apoptosis by releasing IL-1, IL-4, IL-6, IL-8, TNF-α, IFN-γ, TGF-β, MCP-3, MCP-4, leukotriene B4 (LTB4), and chymotrypsin. However, the prognostic value of mast cells in human solid tumors is unclear and controversial. |
| Mononuclear cell | Macrophage | Macrophages are functionally heterogeneous, with M1 macrophages secreting antimicrobial molecules and proinflammatory cytokines, including ROS, NO, and IL-6, as well as maintaining a strong antigen-presenting capacity and inducing a strong Th1 response. M2 macrophages secrete anti-inflammatory cytokines, including TGF-β and IL-10, as well as proteases (e.g., arginase-1 and MPPs) and play a key role in limiting immune responses, inducing angiogenesis and tissue repair. |
|  | Dendritic cell (DC) | Dendritic cells are the most efficient antigen presenting cells and play a key role in both intrinsic and adaptive immunity. Under physiological conditions, extracellular microbial proteins are phagocytosed by DCs and presented to CD4^+^ T cells via MHC-II, and cytoplasmic microbial proteins are presented to CD8^+^ T cells via MHC-I. DCs in the TME play either pro- or anti-cancer roles, depending on the stage of development and tumor staging. |
| Lymphocyte | T cell | Antitumor immune response is primarily orchestrated by CD8^+^ T cells, which differentiate into cytotoxic effector cells (CTLs) upon specific recognition of antigenic peptides displayed on the surface of APCs via MHC-I. In contrast, CD4^+^ T cells, commonly referred to as helper T cells (Th), can both enhance and suppress antitumor immunity. Th1 cells secrete IFN-γ and TNF-α to assist in the destruction of tumors by B cells, NK cells, and CTLs. On the other hand, Th2 cells promote tumor growth by secreting anti-inflammatory mediators, while Tregs are a highly immunosuppressive subset of CD4^+^ T cells. |
|  | B cell | B cells were initially believed to contribute exclusively to tumorigenesis, given that their antibodies can encourage cancer growth and dissemination in certain mouse models. However, more recent research has revealed that tumor-infiltrating B cells (TILs B) are not always destructive. In fact, they can actively participate in the tumor immune response, presenting antigens, producing cytokines, and, when activated, transforming into plasmablasts. In metastatic melanoma, TILs B cells are considered to be the next best predictor of disease prognosis after CTL. |
|  | Natural killer cell (NK cell) | NK cells can function spontaneously without prior sensitization. In addition, they rapidly produce IFN-γ and TNF-α, which mediate an early response to tumors.The immune response of NK cells involves a variety of cytokines (IFN-γ, IL-10, IL-5, IL-13, TNF-α, and GM-CSF) and chemokines (MIP-1α, MIP-1β, IL-8, and CCL5).IFN-γ is secreted by NK through the activation of secreted by its surface receptor NKG2D, which plays a key role in anti-tumor activity. |
| Myeloid-derived suppressor cell（MDSC） | | MDSCs have the capability to restrict the normal intrinsic and adaptive immune functions of the body's immune cells. As for intrinsic immunity, MDSCs suppress the function of NK cells by down-regulating the expression of NKG2D via membrane-bound TGF-β. Moreover, MDSCs stimulate Treg expansion and augment the negative regulatory effects of Treg on immunity [1; 2]. In adaptive immunity, MDSCs can compete for cysteine in the body environment, enhance the activity of iNOS and Arg-1, thereby depleting L-arginine and obstructing T-cell generation. MDSCs can also suppress T-cell immunity by generating ROS. |
