# Supplementary material for: Advances in macrophage and T cell metabolic reprogramming and immunotherapy in the tumor microenvironment
Source: PeerJ. 2024 Jan 15;12:e16825. doi: 10.7717/peerj.16825 (PMC10795528; doi:10.7717/peerj.16825)
Supplement: Supplemental Information 2 [file peerj-12-16825-s002.docx]

Table S2. The current clinical trials of metabolic combined immune-checkpoint agents

| **Pathway** | **Target** | **Drugs** | **Phase** | **NCT number** |
| --- | --- | --- | --- | --- |
| Adenosine | A2AR antagonist | AZD4635 | 1 | NCT02740985 |
|  |  | Ciforadenant | 1 | NCT02655822 |
|  |  | AZD4635 | 2 | NCT04495179 |
|  | CD73 | NZV930 | 1 | NCT03549000 |
|  |  | CPI-006 | 1 | NCT03454451 |
|  |  | Oleclumab | 2 | NCT04668300 |
|  |  |  | 1 | NCT03773666 |
|  |  | BMS-986179 | 1 | NCT02754141 |
|  |  | MEDI 9447 | 2 | NCT03267589 |
| Arginine | Arginine deiminase | ADI PEG20 | 1 | NCT03254732 |
|  |  |  | 1 | NCT03922880 |
|  | Arginase inhibitor | INCB001158 | 1 | NCT02903914 |
| Gene Therapy | DNA plasmid-IL-12 | TAVO | 2 | NCT03823131 |
| Glucose | Glycolysis | Metformin | 2 | NCT03048500 |
|  |  |  | 2 | NCT03800602 |
|  |  |  | 2 | NCT03994744 |
|  |  |  | 1 | NCT03311308 |
| Glutamine | GLS1 inhibitors | CB-839 | 1 | NCT02771626 |
| Lower Treg | Anti-TNFRSF4 | MEDI 0562 | 2 | NCT03267589 |
| PTGS | EP4 antagonist | grapiprant | 1 | NCT03658772 |
|  | COX inhibitors | Acetylsalicylic Acid | 1 | NCT03245489 |
| Tryptophan | IDO inhibitor | Indoximod | 1 | NCT02073123 |
|  |  |  | 2 | NCT03301636 |
|  |  | GDC-0919 | 1 | NCT02471846 |
|  | IDO1 inhibitor | Epacadostat | 2 | NCT03006302 |
|  |  |  | 2 | NCT04586244 |
|  |  |  | 2 | NCT03823131 |
|  |  |  | 2 | NCT04463771 |

GLS1, Glutaminase 1; IDO, Indoleamine 2,3-dioxygenase; A2AR, Adenosine receptors; PTGS, prostaglandin-endoperoxide synthase; EP4, Prostaglandin E2 receptor 4; COX, cyclo-oxygenase.
